# Protein Kinases C-Mediated Regulations of Drug Transporter Activity, Localization and Expression

**DOI:** 10.3390/ijms18040764

**Published:** 2017-04-04

**Authors:** Abdullah Mayati, Amélie Moreau, Marc Le Vée, Bruno Stieger, Claire Denizot, Yannick Parmentier, Olivier Fardel

**Affiliations:** 1 Institut de Recherches en Santé, Environnement et Travail (IRSET), UMR INSERM U1085, Faculté de Pharmacie, 2 Avenue du Pr Léon Bernard, 35043 Rennes, France; abdullah.mayati@univ-rennes1.fr (A.M.); marc.levee@free.fr (M.L.V.); 2 Centre de Pharmacocinétique, Technologie Servier, 25–27 Rue Eugène Vignat, 45000 Orléans, France; amelie.moreau@servier.com (A.M.); claire.denizot@servier.com (C.D.); yannick.parmentier@servier.com (Y.P.); 3 Department of Clinical Pharmacology and Toxicology, University Hospital Zurich, University of Zurich, Rämistrasse 100, 8091 Zurich, Switzerland; bruno.stieger@uzh.ch; 4 Pôle Biologie, Centre Hospitalier Universitaire, 2 rue Henri le Guilloux, 35033 Rennes, France

**Keywords:** protein kinases C, drug transporters, hepatocytes, multidrug resistance, pharmacokinetics

## Abstract

Drug transporters are now recognized as major actors in pharmacokinetics, involved notably in drug–drug interactions and drug adverse effects. Factors that govern their activity, localization and expression are therefore important to consider. In the present review, the implications of protein kinases C (PKCs) in transporter regulations are summarized and discussed. Both solute carrier (SLC) and ATP-binding cassette (ABC) drug transporters can be regulated by PKCs-related signaling pathways. PKCs thus target activity, membrane localization and/or expression level of major influx and efflux drug transporters, in various normal and pathological types of cells and tissues, often in a PKC isoform-specific manner. PKCs are notably implicated in membrane insertion of bile acid transporters in liver and, in this way, are thought to contribute to cholestatic or choleretic effects of endogenous compounds or drugs. The exact clinical relevance of PKCs-related regulation of drug transporters in terms of drug resistance, pharmacokinetics, drug–drug interactions and drug toxicity remains however to be precisely determined. This issue is likely important to consider in the context of the development of new drugs targeting PKCs-mediated signaling pathways, for treating notably cancers, diabetes or psychiatric disorders.

## 1. Introduction

Mammalian drug transporters are integral membrane proteins mediating active transport or facilitated diffusion of exogenous and endogenous compounds across cellular membranes, especially plasma membranes. Transport systems are implicated in intestinal absorption, passage across blood–tissue barriers and hepatobiliary or renal elimination of drugs [1,2]. Drug transporters are consequently recognized as playing a major role in drug disposition and, beyond, in drug efficacy and toxicity as well as in pharmacokinetic drug–drug interactions [3,4,5]. The study of the potential interactions of new molecular entities with the main drug transporters is thereby now recommended by drug regulatory agencies [6]. Besides, some drug transporters directly control anticancer drug accumulation in cancer cells and, in this way, sensitivity to chemotherapeutic agents [7].

**Figure 1:**
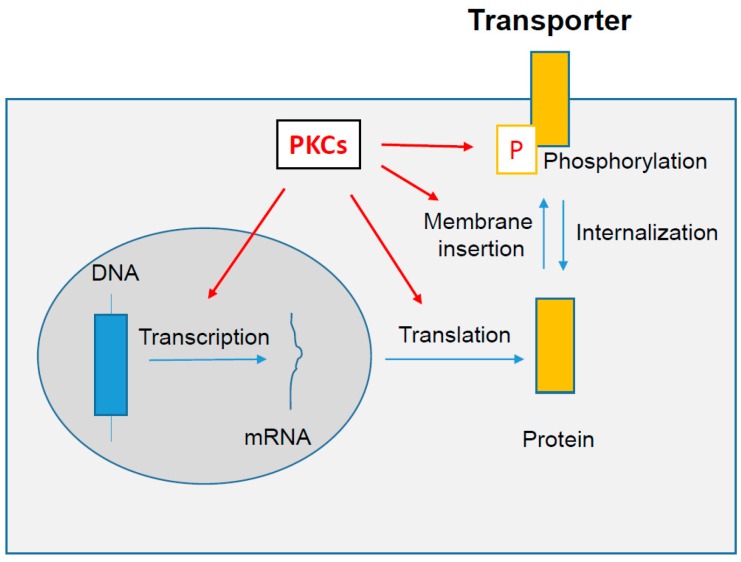
Schematic representation of putative cellular targets of protein kinases C (PKCs, in red) with respect to regulation of drug transporter activity, localization and/or expression. Arrows in red indicate the putative effects of PKCs on transporter regulatory pathways. Arrows in blue correspond to the different transporter processing steps, from gene (DNA) to activity regulation at the plasma membrane by phosphorylation (P, in red). DNA: deoxyribonucleic acid; mRNA: messenger ribonucleic acid.

Various factors, including hormones, cytokines, drugs and environmental contaminants, can modulate expression, localization and/or activity of drug transporters [8,9,10,11,12]. It is important to precisely characterize such regulations, owing notably to the major role played by transporters in pharmacokinetics and some toxic effects of drugs. They may implicate various signaling pathways, including those linked to serine/threonine protein kinases C (PKCs). Indeed, PKCs-mediated phosphorylation of some drug transporters has been shown to directly control their activity [13,14]. Activation of PKCs also modulates drug transporter localization in plasma membrane of polarized cells such as hepatocytes [15,16]. PKC activation additionally impairs messenger ribonucleic acid (mRNA) and/or protein levels of drug transporters [17,18]. As graphically summarized in Figure 1, PKCs can thus theoretically affect different aspects of drug transporter regulation, e.g., transcriptional or translational mechanisms controlling transporter expression, membrane insertion or internalization processes and phosphorylation status of transporters.

PKCs play an important, although often complex, role in various diseases, including cancer, cardiovascular dysfunctions, psychiatric pathologies and metabolic disorders like diabetes [19,20]. PKCs are consequently considered as potential attractive therapeutic targets [21]. Therefore, the search for PKC inhibitors is an active area of drug development [22] and it may be hypothesized that the clinical use of such novel chemical entities may directly or indirectly affect PKCs-mediated regulation of drug transporters. Moreover, PKCs can be directly activated by some drugs, like ingenol mebutate (a drug used for the treatment of actinic keratoses, also known as ingenol 3-angelate or PEP005) [23]. Such PKCs-activating drugs may therefore trigger PKCs-related transporter regulation. PKCs-related regulatory ways for drug transporters are therefore important to be considered. In the present review, we summarize the reported effects of PKCs on drug transporter activity, localization and expression. Furthermore, we discuss the possible clinical consequences of such PKCs-related regulations of membrane transporters in response to physiological or pharmacological effectors, including PKC inhibitors.

## 2. The Drug Transportome

The drug transportome can be defined as the set of membrane transporters handling drugs. Such transporters are usually expressed at the plasma membrane and are grouped into two classes, the solute carriers (SLC) and the ATP-binding cassette (ABC) transporters. SLC transporters behave as influx or efflux transporters, through mediating facilitated diffusion or secondary active transport (co-transport or anti-port), whereas ABC drug transporters act as ATPase-based primary active drug efflux pumps [2].

SLC transporters constitute a large super family of transporters, currently comprising over 400 members organized into 52 families [24,25]. Many of these SLC transporters however await being functionally characterized [26]. The main SLC drug transporters are presented in Table 1. They are usually expressed in organs implicated in drug absorption, metabolism and elimination such as the intestines, liver and kidney. Some of them are also present at blood–tissue barriers, notably at the blood–brain barrier [27].

Transport of anionic drugs is notably assumed by the organic anion transporting polypeptide (OATP, *SLCO*) (protein name, gene name) family [28], that comprises in humans eleven members, including OATP1A2 (*SLCO1A2*), ubiquitously expressed, and OATP1B1 (*SLCO1B1*), OATP2B1 (*SLCO2B1*) and OATP1B3 (*SLCO1B3*), present at the sinusoidal pole of hepatocytes where they mediate uptake of drugs such as statins into the liver. OATP2B1 is also a key actor of the intestinal transport system [29]. The *SLC22A* family comprises (1) organic cation transporters (OCTs), like the uptake transporters OCT1 (*SLC22A1*), expressed at the sinusoidal pole of hepatocytes, and OCT2 (*SLC22A2*) [30], present on the basolateral pole of renal proximal tubule cells, respectively; (2) organic anion transporters (OATs), such as the renal OAT1 (*SLC22A6*), OAT3 (*SLC22A8*) and OAT4 (*SLC22A11*), and the sinusoidal hepatic OAT2 (*SLC22A7*) [31]; and (3) organic cation/carnitine transporter (also known as organic cation transporter novel) (OCTN) 1 (*SLC22A4*) and OCTN2 (*SLC22A5*), sharing numerous substrates with OCTs [32]. The *SLC47A* family corresponds to multidrug and toxin extrusion transporters (MATEs) present at the apical pole of hepatocytes (MATE1/*SLC47A1*) and renal proximal tubule cells (MATE1 and MATE2-K/*SLC47A2*), where they act as H^+^/organic cation antiporters for putatively secreting drugs into bile or urine [33]. Additional main SLC transporters handling drugs correspond to the hepatic sodium-taurocholate co-transporting polypeptide (NTCP/*SLC10A1*), that can mediate statin transport [34], and proton-coupled peptide transporters (PEPTs/*SLC15A*) PEPT1 (*SLC15A1*) and PEPT2 (*SLC15A2*) [35], notably located in intestine and kidney, respectively, as well as nucleoside transporter proteins [36]. These nucleoside transporters handle a variety of nucleoside-derived drugs, mostly used in anticancer or antiviral therapy. They are split into two families, i.e., the sodium-dependent concentrative nucleoside transporters (CNT/*SLC28A*) containing three members (CNT1/*SLC28A1*, CNT2/*SLC28A2* and CNT3/*SLC28A3*) and the equilibrative nucleoside transporters (ENT/*SLC29A*), containing four members, notably ENT1/*SLC29A*1 and ENT2/*SLC29A2*.

Classification of the main ABC drug transporters, which belong to 3 of the 7 families of ABC transporters, is shown in Table 1. The historically first identified mammalian ABC drug transporter was P-glycoprotein (P-gp), encoded by multidrug resistance gene 1 (*MDR1/ABCB1*) and conferring multidrug resistance by expelling a wide range of structurally unrelated anticancer drugs out of cancer cells [7,37,38]. P-gp also transports a lot of non-anticancer drugs like digoxin. It is physiologically expressed in absorptive or excretory organs such as the gut, the liver and the kidney [39]. P-gp is also present at various blood–tissue barriers. It is thus expressed at the luminal pole of brain capillary endothelial cells and prevents the entry of drugs into brain by actively expelling them into the blood stream [40]. In this way, P-gp contributes to the blood–brain barrier in a major way [41]. Multidrug resistance-associated protein (MRP) 1 (*ABCC1*) is another ABC transporter implicated in cancer multidrug resistance. MRP1 exhibits a broad tissue distribution and handles a wide range of xenobiotics, including anionic drugs and drug conjugates [42]. Other members of the MRP/*ABCC* family expelling drugs from cells include (1) MRP2 (*ABCC2*), sharing numerous substrates with MRP1, and expressed in many epithelia and at the canalicular pole of hepatocytes [43]; (2) MRP3 (*ABCC3*), present at the sinusoidal pole of hepatocytes where it transports xenobiotics from the liver to blood for secondary renal elimination; (3) MRP4 (*ABCC4*) also expressed at the sinusoidal pole of hepatocytes, but additionally in kidney and at blood–brain barrier, and having a wide substrate specificity, including nucleoside analogues and antiviral drugs [44], and (4) MRP5 (*ABCC5*), almost ubiquitously expressed in humans and exporting a broad range of natural and xenobiotic compounds such as cyclic guanosine monophosphate, antiviral agents and chemotherapeutic drugs [45]. Like P-gp and MRP1, the ABC transporter breast cancer resistance protein (BCRP/*ABCG2*) transports both anticancer drugs and non-anticancer drugs and is found at blood–tissue barriers and in the gut and excretory organs like the liver and kidney [46]. The bile salt export pump (BSEP/*ABCB11*), almost exclusively expressed at the canalicular pole of hepatocytes, plays an important role in bile salt secretion into bile [47] and in vitro can transport rosuvastatin [48].

## 3. The Protein Kinases C (PKCs) Family

The mammalian PKC family comprises 10 members that represent the products of nine different genes located in different chromosomes. These PKC isozymes have been classified into three groups: (1) “conventional” or “classical” PKCs (cPKCs) that are composed of PKCα, two splice variants of PKCβ (PKCβI and PKCβII) and PKCγ; (2) “novel” PKCs (nPKCs), a group that includes PKCδ, PKCε, PKCη and PKCθ; and (3) “atypical” PKCs (aPKCs) ζ and ι/λ (PKCι is found only in primates and PKCλ is its mouse counterpart) (Table 2) [49]. The protein kinase D1 was additionally initially considered as a PKC isoform under the name of PKCµ [50], before being definitively classified as a novel subgroup of the calcium/calmodulin-dependent protein kinase family [51]. While some PKC isoforms are expressed in a tissue-specific manner, i.e., PKCθ is expressed primarily by skeletal muscle, lymphoid organs, and hematopoietic cell lines and PKCγ is detected largely in human neuronal tissues, most PKC isoforms are ubiquitous. Moreover, many cells coexpress multiple PKC family members [52]. Like many other protein kinases, PKCs have a regulatory region and a catalytic region [53]. cPKCs and nPKCs are activated by diacylglycerol (DAG), a lipid-derived second messenger that is transiently generated upon activation of phospholipase C following stimulation of membrane receptors such as tyrosine-kinase and G-protein-coupled receptors [54]. DAG activates cPKCs and nPKCs through binding to the C1 domain of the regulatory region of these PKCs. Activation of cPKCs, known as calcium-sensitive, additionally requires the binding of calcium to the C2 domain of their regulatory region [53]. aPKCs display unique regulatory properties: they are unable to bind DAG or calcium and rather depend on protein-protein interactions and phosphorylation for their activation [55].

Phorbol esters such as phorbol-12-myristate-13-acetate (PMA) mimic the effects of DAG [56]. They bind to the C1 region of cPKCs and nPKCs and by this way directly and potently activate them. It is noteworthy that PMA can be considered as a reference activator of PKCs and its main effects on drug transporter activity, localization and expression are consequently summarized in Table 3.

Upon physiological or pharmacological activation, PKCs usually translocate from the cytosolic (soluble) fraction to the cell particulate fraction, which includes the plasma membrane as well as many other cellular organelles, including mitochondria, Golgi, endoplasmic reticulum and nuclear membrane. PKCs primarily trigger their biological effects through phosphorylating serine/threonine sites of their substrates, which may have diverse biological roles and/or may downstream activate other signal transduction pathways. In this way, PKC activation is thought to regulate many cellular functions, including cell proliferation and cell death, gene transcription and translation, alteration of cell morphology and cell migration, regulation of ion channels and receptors, cell–cell contact and cell polarity [19,20,21,53,57,58].

## 4. PKCs-Dependent Regulation of Drug Transporter Activity

Drug transporters, as many proteins, usually contain several consensus phosphorylation sites, which may be, at least for some of them, targeted by PKCs. This may in turn affects drug transport activity.

P-gp has thus been described as a phosphoglycoprotein, i.e., the pump can be phosphorylated on serine residues [76] by PKCs, thus establishing a link between PKCs and multidrug resistance [77]. Indeed, PKC activators such as PMA increase P-gp phosphorylation [78], which generally results in enhanced activity of the efflux pump in human cancer cell lines [13] and thus multidrug resistance [59,60]. The ubiquitination/degradation of the transporter is however not affected [79]. The fact that human multidrug resistant cancerous cell lines overexpress PKCs, which likely potentiates P-gp phosphorylation, has consequently been hypothesized as contributing to drug resistance [80]. This highlights the interest of modulating PKC activity for reversing resistance [81,82]. PMA also enhances P-gp activity in normal cells/tissues such as isolated mouse proximal tubule segments [61]. The sites of P-gp phosphorylated by PKCs are thought to correspond to Ser661 and Ser671, and one or more of Ser667, Ser675, and Ser683 [83]. Different PKC isoenzymes are thought to be involved in these phosphorylations, notably the cPKCs α, βI, βII and γ, the nPKCs δ, ε and η as well as the aPKC ζ [84]. Some of these PKC isoenzymes, including cPKCs, but not PKCδ, have been additionally shown to physically interact with P-gp in co-immunoprecipitation assays in human cancer cells [85]. Among PKC isoforms, PKCα may play a major role because specifically targeting this PKC isoform through chemical inhibition or transcriptional suppression permits attenuation or reversal of drug resistance of human cancer cell lines [86,87,88,89], whereas its overexpression enhances P-gp phosphorylation and multidrug resistance [90,91].

It is however noteworthy that the link between PKC activity and P-gp-mediated drug resistance has been challenged [92]. Indeed, the major P-gp phosphorylation sites are in fact located within the linker region, not directly implicated in transport activity [93]. Moreover, various PKC inhibitors like the pan-PKC inhibitors staurosporine and chelerythrine, the PKCβ inhibitor enzastaurin and the bisindolylmaleimide (BIM) PKC inhibitors GF 109203X (also known as BIM-I or Gö 6850) and Ro 32-2241 can suppress drug resistance by directly binding to and inhibiting P-gp, independently of P-gp phosphorylation [94,95,96,97]. The findings that bryostatin 1, which represses PKC expression, failed to reverse multidrug resistance of human cancer cells [98], and that PMA decreased P-gp activity in teleost renal proximal tubules [99], is conflicting with the assertion that PKC activity is positively correlated with P-gp-mediated transport, or, at least, suggests that it has to be relativized according to the cell type and/or the species [100]. Moreover, at the blood–brain barrier, activation of the PKCβ1 isoform rapidly decreases P-gp activity and enhances drug delivery to the rat brain [101]. Certain St. John’s Wort constituents, especially quercetine, also down-modulate P-gp transport activity in porcine brain capillaries in a PKCs-dependent manner [102]. Finally, a PMA-mediated decrease in drug accumulation into human cancer cells has been postulated to occur in a P-gp-independent manner [103].

MRP2 transport activity is likely to be also regulated by PKCs. For example, MRP2 could be phosphorylated by cPKCα and nPKCϵ, thus enhancing MRP2 activity, which contributes to the anticholestatic effect of tauroursodeoxycholic acid, the taurine conjugate of ursodeoxycholate, in rat liver [104]. Some physiological effectors such as endothelin-1 may additionally regulate MRP2 activity via PKC activity in shark rectal salt gland tubules [105]. By contrast, activities of other MRPs, as well as those of BCRP and BSEP, have not been formally demonstrated to be influenced by PKCs-mediated phosphorylation. As for P-gp activity, that of BCRP was inhibited by the PKCβ inhibitor enzastaurin in human cancer cell lines, probably via PKCs-independent reduction of BCRP ATPase activity [95]. Similarly, various PKC inhibitors belonging to the chemical family of BIMs blocked BCRP-mediated transport in a PKC-unrelated manner [106]. The efficient modulation of MRP1-mediated drug resistance by the PKC inhibitor GF 109203X [107] is also probably due to direct interaction of this PKC inhibitor with MRP1 and not to hypothetical alteration of PKC-dependent alteration of MRP1 phosphorylation.

Among SLC drug transporters, OCT1, as well as OCT2, possesses several potential PKC phosphorylation sites in the intracellular loops [108]. PKC-phosphorylation sites moreover determine substrate selectivity and transport regulation for rat OCT1 [109]. PKC activation by PMA however failed to stimulate activity of human OCT1 and OCT2 [108,110]. Whether PMA may modulate transport mediated by other organic cation transporters such as MATE1 and MATE2-K is not known. Activity of these MATE transporters can however be blocked by the BIM Ro 31-8220 (also known as BIM-IX), that also inhibits that of OCT1 in a PKC-independent manner in human OCT1-transfected HEK293 cells, whereas that of OCT2 is *cis*-stimulated [111]. Among SLCs handling organic anions such as OATPs and OATs, OATP1B3, that mediates hepatic uptake of various drugs and endogenous compounds, has been demonstrated to be phosphorylated by PKCs; such a post-translational regulation results in decreased OATP1B3 transport activity in primary human hepatocytes in response to PMA [14]. The phorbol ester also reduces rat OATP-mediated transport in transfected Xenopus laevis oocytes [112] and OAT3 activity in isolated rabbit renal proximal tubules [70], suggesting that this OAT constitutes a target for PKCs. For nucleoside transporter proteins, ENT1 can be phosphorylated by PKCs at multiple sites [113]. At least phosphorylation at Ser281 increases ENT1 activity in pig kidney epithelial nucleoside transporter deficient (PK15-NTD) cells transfected with human ENT1 [73]. Otherwise, various PKC inhibitors such as GF 109203X, Ro 31-8220 and arcyriarubin A (also known as BIM-IV) act as potent inhibitors of ENT1 [114]. The BIM Ro 31-6045, a staurosporine analog that does not inhibit PKCs, also blocks ENT1 and ENT2 [114,115], suggesting that ENT1/2 inhibition may be shared by BIMs, irrespective of PKC inhibition. Peptide transport by PEPT1 and PEPT2 in human intestinal Caco-2 cells and porcine renal LLC-PK1 cells has finally been demonstrated to be down-regulated by PMA [74,75]. Such data support the idea that PEPT1 and PEPT2 activity may be directly modulated by PKCs-mediated phosphorylation of these transporters.

## 5. PKCs-Dependent Regulation of Drug Transporter Localization

Plasma membrane localization of drug transporters is a prerequisite for drug transporter activity. Moreover, in polarized epithelial cells such as hepatocytes, proximal tubular cells or enterocytes, drug transporters have to be targeted to the correct location, e.g., the canalicular membrane of hepatocytes for P-gp, MRP2 and BSEP. Among PKC isoforms, aPKCs play a major role in the polarization process of epithelial cells through the partitioning-defective (PAR)-aPKC polarity complex [116,117], and may thus be considered as indirectly governing polarized expression of drug transporters. Trafficking between intracellular vesicles and plasma membrane, including endocytosis and recycling steps, also contributes to polarization [118]. This occurs for various SLC and ABC drug transporters [119,120,121,122,123] and may be a notable method of post-translational regulation of drug transporter function, even if in vivo relevance in physiological situations remains to be fully established. Such trafficking has been demonstrated to constitute a target for PKCs, which, in this way, can control transporter activity.

**Figure 2:**
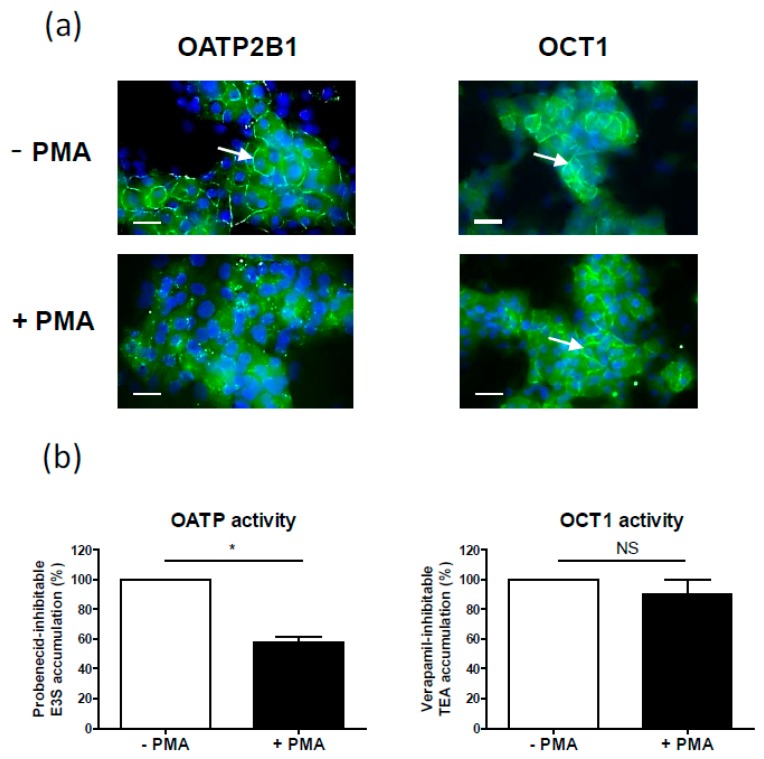
Effect of the protein kinase C (PKC) activator phorbol-12-myristate-13-acetate (PMA) on (**a**) organic anion transporting polypeptide (OATP) 2B1 and organic cation transporter (OCT) 1 localization (**a**) and activity (**b**) in human highly-differentiated hepatoma HepaRG cells. Human HepaRG cells were exposed or not to 100 nM PMA for 1 h. (**a**) OATP2B1 and OCT1 expression were next analyzed by immunofluorescence as previously reported [129]. Green fluorescence corresponds to transporter immunolabeling, whereas blue fluorescence reflects 4,6-diamidino-2-phenylindole-stained nuclei. Arrows indicate transporter-related sinusoidal membrane fluorescence. Bar = 10 µm; (**b**) Probenecid-inhibitable uptake of estrone-3-sulfate (E3S), reflecting OATP activity [124], and verapamil-inhibitable uptake of tetraethylammonium (TEA), reflecting OCT1 activity [124], were determined as previously described [129]. Data are expressed as % of transporter activity found in cells not exposed to PMA, arbitrarily set at 100%. They are the means ± standard errors of the means (SEM) of at least three independent assays. * *p* < 0.05 and NS, not statistically significant (Student’s *t* test).

The PKC activator agent PMA has thus been shown to stimulate internalization of OATP transporters such as OATP1A2, OATP1B1 and OATP2B1 in cultured cells [66,67,68]. As an example, cell surface expression of OATP2B1 at the sinusoidal pole of human highly-differentiated hepatoma HepaRG cells was markedly decreased in response to a short 1-h treatment with 100 nM of PMA (Figure 2a). In parallel, OATP transport activity, determined through measuring probenecid-inhibitable uptake of estrone-3-sulfate [124], was significantly reduced by PMA (Figure 2b). Co-treatment by the pan-PKC inhibitor Gö6983 fully prevented PMA-mediated decreased of OATP activity (data not shown), thus confirming that PMA effect towards OATP2B1 was related to PKC activation. By contrast, the phorbol ester failed to alter sinusoidal membrane expression of OCT1 in HepaRG cells (Figure 2a); it concomitantly did not impair verapamil-inhibitable uptake of tetraethylammonium (Figure 2b), which corresponds to OCT1 activity [124]. Such data therefore demonstrated that localization of OCT1 transporter was not regulated by PKCs in hepatic cells. PKC-triggered OATP1A2 internalization in COS-7 cells transfected with OATP1A2 was blocked by the cPKC inhibitor Gö6976 and was dependent on clathrin-dependent endocytosis, but not on the caveolin-dependent pathway [66]. Similarly, OATP2B1 internalization caused by PMA was related to clathrin-mediated endocytosis, followed by lysosomal degradation in OATP2B1-transfected MDCKII cells [68], whereas internalized OATP1B1 co-localized with early and recycling endosomal markers in OATP1B1-transfected HEK293 cells [67]. Activation of PKCs by PMA also results in altered trafficking of the ENT1 nucleoside transporter, with significant increase in the plasma membrane localization of ENT1 [73]. Similarly, PKC activation inhibits OAT1 activity by promoting ubiquitination of the transporter in OAT1-transfected COS-7 cells, which then leads to an accelerated internalization of the transporter from cell surface to intracellular compartments in response to PMA [69]. The PKC isoform PKCα is responsible for this OAT1 ubiquitination [125] and is also involved in angiotensin II-induced retrieval of OAT1 and OAT3 from the plasma membrane [126,127]. By contrast, PKCζ activation leads to increased OAT1 and OAT3 activity in rodent renal cortical slices, which may result, at least for OAT3, from increased trafficking into the plasma membrane [128]. With respect to the carnitine transporter OCTN2, its presence at the cell surface, as well as its activity, have been shown to be enhanced by PMA in cultured rat astrocytes, thus supporting the idea of a multi-protein complex regulated by PKCs and implicated in OCTN2 trafficking to the cell surface [72].

**Figure 3:**
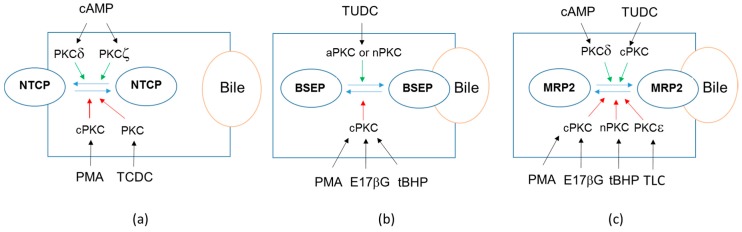
Schematic representation of protein kinase C (PKC) effects on trafficking of (**a**) sodium-taurocholate co-transporting polypeptide (NTCP), (**b**) bile salt export pump (BSEP) and (**c**) multidrug resistance-associated protein (MRP) 2 in hepatocytes. Black arrows indicate activation of PKCs by compounds. Red arrows indicate stimulation of transporter internalization by PKCs. Green arrows show stimulation of membrane insertion of transporters by PKCs. Blue arrows correspond to trafficking (internalization/membrane insertion) of transporters. PMA: phorbol-12-myristate-13-acetate; TCDC: taurochenodeoxycholate; E17βG: estradiol 17β-d-glucuronide; tBHP: tertio-butylhydroperoxide; TLC: taurolithocholate; cAMP: 3′,5′-cyclic adenosine monophosphate; TUDC: tauroursodeoxycholate

PKCs also play a major, but complex, role in plasma membrane location of transporters involved in bile salt transport, i.e., NTCP, BSEP and MRP2. They are closely associated with bile formation, and beyond, with cholestatic or choleretic effects of endogenous or exogenous compounds, as recently reviewed [16,130]. A schematic overview of PKC effects towards bile salt transporters in hepatocytes is depicted in Figure 3.

For the sinusoidal bile salt uptake transporter NTCP (Figure 3a), PMA stimulates its endocytosis in primary rat hepatocytes and in NTCP-transfected hepatoma HepG2 cells. This internalization implicates cPKCs and is likely involved in the cholestatic effect of the phorbol ester [64,71]. Similarly, PMA reduces plasma membrane content of the ileal apical sodium-dependent bile acid transporter (ASBT/*SLC10A2*), suggesting modulation by vesicular recycling [131]. PKCs are also involved in NTCP internalization caused by the bile acid taurochenodeoxycholate in rodent liver [132]; the exact nature of the implicated PKC isoform(s) remains however to be characterized. The nPKCδ as well as the aPKCζ are involved in cyclic adenosine monophosphate (cAMP)-mediated stimulation of NTCP translocation to the plasma membrane in primary rat hepatocytes [15] or NTCP-transfected human hepatoma HuH-7 cells [133]. Plasma membrane localization rather than kinase activity of PKCδ may however be involved in cAMP-induced NTCP translocation [134], whereas PKCζ is required for microtubule-based motility of vesicles containing NTCP [135].

With respect to BSEP (Figure 3b), its localization at the apical membrane of isolated rat hepatocytes or NTCP-transfected human hepatoma HepG2 cells is stimulated by the choleretic agent tauroursodeoxycholate in a PKC-dependent manner [136]. This effect was however not inhibited by the selective cPKC inhibitor Gö6976, thus suggesting that it implicates nPKC or aPKC isoform(s) [136]. Such data therefore fully support the hypothesis that ursodeoxycholate conjugates may improve impaired bile secretion of the cholestatic liver by stimulating insertion of carrier proteins into the canalicular hepatocyte membrane [137,138]. BSEP may therefore be considered as a potential therapeutic target [139]. cPKCs can however also trigger BSEP internalization, which likely contributes in a major way to cholestatic effects of PMA and of thymeleatoxin, a selective activator of cPKCs, in rat liver [64]. These Ca^2+^-dependent PKC isoforms are additionally involved in oxidative stress-triggered retrieval of BSEP from canalicular membrane in isolated rat hepatocyte couplets [140]. Similarly, they are implicated in BSEP internalization in response to the cholestatic agent estradiol 17β-d-glucuronide in rat hepatocytes [141].

For MRP2 (Figure 3c), cPKCs, notably the isoform PKCα, are additionally implicated in MRP2 internalization due to estradiol 17β-d-glucuronide, thus pointing out their major role in cholestasis caused by some estrogenic metabolites [141]. The implication of PKCs in MRP2 retrieval from the apical membrane is also supported by the fact that PMA triggers PKC-dependent redistribution of MRP2 from the apical membrane to its basolateral counterpart in human HepG2 cells [65]. By contrast, MRP3 localization as well as its activity are not impaired. In the same way, the selective cPKC activator thymeleatoxin reduces apical localization of MRP2 in rat intestine through modulating the protein–protein interaction between MRP2 and ezrin [142], which serves as an intermediate between the plasma membrane and the actin cytoskeleton. Ezrin additionally appears to regulate membrane expression of MRP2 and also of P-gp in human intestinal Caco-2 cells [143]. PKCα, but also PKCδ and PKCε, can in fact directly stimulate ezrin Thr567 phosphorylation, which in turn results in reduced expression of MRP2 at the apical membrane of hepatocytes [144]. Such PKC/ezrin-dependent regulation of MRP2 localization may be responsible for MRP2 internalization during human obstructive cholestasis [144]. In addition to ezrin, radixin, a cytoskeletal protein linking MRP2 to F-actin, is involved in MRP2 internalization in rat hepatocytes in response to the oxidative agent tertio-butylhydroperoxide, which implicates one nPKC isoform that remains to be formally identified [145]. The nPKCε has additionally been shown to mediate MRP2 retrieval from the apical membrane in response to the cholestatic agent taurolithocholate through phosphorylating myristoylated alanine-rich C kinase substrate in human NTCP-transfected hepatoma HuH-7 cells [146]. It is however noteworthy that the effects of PKCs on MRP2 localization are rather complex and may notably depend on the nature of the stimulus activating PKC or of additional signaling ways. Indeed, nPKCδ, whose overexpression stimulates MRP2 internalization through ezrin-dependent mechanism as described above, may also trigger MRP2 translocation to the apical membrane of rat hepatocytes, notably in response to cAMP, which primarily activates this nPKC isoform [15]. In the same way, tauroursodeoxycholate inserts MRP2 into canalicular membranes and stimulates organic anion secretion by PKC-dependent mechanism in rat cholestatic liver [147]. Among PKC isoforms, PKCα is likely to be implicated in these anticholestatic effects of tauroursodeoxycholate [104]. Therefore, this PKC isoform may prevent (membrane insertion of MRP2) or stimulate (MRP2 internalization, notably in response to estradiol 17β glucuronide, as reported above) cholestasis, according to the initial stimulus leading to PKCα activation.

## 6. PKCs-Dependent Regulation of Drug Transporter Expression

Besides localization, expression levels of various drug transporters have been shown to be regulated by PKCs.

In human tumoral cell lines, P-gp expression is usually increased by exposure to PMA and other PKC-activating agents [17,62,63], in a PKC-dependent manner and independently of mitogen activated protein kinase (MAPK) signaling pathways [148]. Such an up-regulation implicates transcriptional activation of *MDR1* promoter activity [149]. Among PKC isoforms, PKCε as well as PKCα and PKCθ are likely implicated in P-gp up-regulation. Indeed, PKCε activation mediates the induction of P-gp in cultured cancer prostate cells [150], whereas the *MDR1* promoter has been shown to be regulated PKCα and PKCθ [151]. Moreover, silencing PKCα by RNA interference increased drug sensitivity of ovarian cancer cells through decreasing P-gp levels [152]. PKCα activation is also associated with induced P-gp expression in non-cancerous tissues such as the liver of diabetic rats, suggesting a link between hyperglycemia and P-gp overexpression via PKC [153]. *MDR1/ABCB1* mRNA expression has additionally been shown to be transiently induced by PMA in primary human hepatocytes [18]. However, inhibition of PKCα isoform enhances P-gp expression and the survival of cultured LoVo human colon adenocarcinoma cells to doxorubicin exposure [154]. Such data, that are rather contradictory with those discussed above, suggest that the exact nature of the effects of PKCα towards P-gp expression may depend on the cell type.

Expression of other transporters has been shown to be regulated by PKCs. Indeed, induction of MRP1 and MRP2 mRNA levels by the anticancer drug doxorubicin alone or associated to the fibroblast growth factor 2 is inhibited by the PKC inhibitor chelerythrin in cultured rat cardiomyocytes, thus indicating that it depends on PKC activity [155]. ENT1 suppression by high glucose in rat cardiac fibroblasts is mediated by aPKCζ [156]. Finally, PMA treatment of primary human hepatocytes, that induces *MDR1*/*ABCB1* mRNA expression as reported above, concomitantly reduces those of OATP1B1, OATP1B3, OATP2B1, NTCP, OCT1 and BSEP and enhances that of MRP3, without impairing those of MRP2 and BCRP [18]. Such PKCs-dependent changes in transporter expression have been hypothesized to be linked to epithelial–mesenchymal transition triggered by PKC activation in hepatic cells like human hepatoma HepaRG cells [18].

## 7. Putative Clinical Relevance of PKCs-Related Alteration of Transporter Activity, Localization and/or Expression

The exact clinical relevance of the multiple, and sometimes opposite, effects of PKCs on drug transporter activity, localization and expression constitutes likely an important issue to consider. Indeed, PKCs are activated in a large set of physiological and pathological signaling pathways related, for example, to hormone effects, cell growth, immune response and cancer progression [21,157]. Such PKCs-activating situations may thus be susceptible to regulating in vivo drug transporter functions. Moreover, PKCs represent potential therapeutic targets for various diseases, including cancers, diabetes, immune disorders and psychiatric pathologies [21,158]. In addition, inhibitors of PKCδ can be used therapeutically to reduce irradiation- and chemotherapy-induced toxicity [159]. There is consequently an increasing number of new molecular entities that target PKCs and have entered clinical trials [22], some of which are listed in Table 4. Such drugs can be hypothesized to interfere with PKCs-related drug transporter regulation, and in this way, may cause potential drug–drug interactions. Such a kinase modulation-based alteration of pharmacokinetics has been recently reported for the tyrosine kinase inhibitor dasatinib and renal OCT2 [160]. The in vivo demonstration of alteration of transporters-related pharmacokinetics due to PKC activity regulation by physiological or pathological effectors or by drugs is however still lacking. In the same way, the exact implication of PKCs in clinical multidrug resistance of cancer cells through putative regulation of ABC transporters like P-gp remains to be precisely determined. Moreover, the effects of PKCs towards drug transporters are rather complex; they may additionally vary, or even antagonize, according to the nature of the incriminated PKC isoform. Such PKC isoform-dependent regulations of transporters may be consequently difficult to evaluate in vivo, owing notably to the limited specificity of activators or inhibitors of PKC isoforms [54]. Indeed, phorbol esters can activate both cPKCs and nPKCs [56], whereas various PKC inhibitors presented as specifically inhibiting one PKC isoform, such as the PKCδ inhibitor rottlerin [161], can in fact hinder various signaling pathways [162]. In addition, besides interfering with PKCs-related pathways, various PKC inhibitors can directly inhibit drug transporters [111], which complicates the interpretation of their effects on PKC-related drug transporter regulation in terms of pharmacokinetics. Consequently, experimental approaches based on knockout, knockdown, and constitutively active and dominant negative mutants may be useful to establish the in vivo relevance and consequences of putative PKCs-mediated regulations of drug transporters.

The fact that PKC activation can, in vivo, regulate transporter function is nevertheless supported by the cholestatic effects of PMA in rat liver [64]. In the same way, exposure to estrogens, through oral contraceptive administration or pregnancy, is known to clinically induce cholestasis in genetically susceptible women [172], which is likely at least partly related to impaired functional expression of PKCs-regulated bile acid transporters like BSEP [141,173]. This highlights the probable in vivo relevance of PKCs-related regulatory ways for transporter expression and regulation. The fact that PMA can trigger OATP2B1 internalization in isolated human placenta [68] supports this conclusion. In this context, modulation of PKC pathways for treating cholestasis through regulating transporter activity or expression may have to be considered in the future. The fact that PKCs-related insertion of the transporters MRP2 and BSEP into canalicular membrane is likely implicated in beneficial anticholestatic effects of tauroursodeoxycholate agrees with this assertion [147] and fully supports the standard use of ursodeoxycholate for treating intrahepatic cholestasis during pregnancy [174]. In addition, human NTCP plays an important role in the entry of hepatitis B and D viruses into hepatocytes and consequent infection [175]. Thus, PKCs, by regulating NTCP trafficking, may also play an important role in hepatic viral infections and, by this way, may constitute attractive therapeutic targets for preventing or treating viral hepatitis.

## 8. Conclusions

PKCs-related signaling pathways can regulate activity, localization and/or expression of various drug transporters in different types of cells or tissues. These transporter regulations often depend of the nature of the incriminated PKC isoform, as well as that of the initial effector activating PKCs. Their exact clinical relevance in terms of drug resistance, pharmacokinetics and potential drug–drug interactions remain however yet to be established. They are nevertheless most likely involved in some pathological processes, such as cholestasis, through modulation of bile acid transporter insertion at the plasma membrane of hepatocytes. Targeting PKCs-related signaling pathways using chemical PKC activators or inhibitors may therefore constitute an attractive therapeutic approach for treating cholestasis. Various other major diseases, including cancers and diabetes, for which PKCs play a crucial role, also represent potential targets for PKC inhibitors. In the context of development of PKCs-interfering drugs, extensive characterization of PKCs-related ways of transporter regulation in terms of pharmacokinetics and potential toxicity deserves further studies. The fact that drugs acting as PKC inhibitors may also impair transporter activity in a PKCs-independent manner through direct interaction with drug binding sites of transporters has however to be kept in mind.

## Figures and Tables

**Table 1:** Classification of main drug transporters. OATP: organic anion transporting polypeptide; NTCP: sodium-taurocholate co-transporting polypeptide; PEPT: peptide transporter; OCT: organic cation transporter; OCTN: organic cation transporter novel; OAT: organic anion transporter; CNT: concentrative nucleoside transporter; ENT: equilibrative nucleoside transporter; MATE: multidrug and toxin extrusion transporter; P-gp: P-glycoprotein; BSEP: bile salt export pump; MRP: multidrug resistance-associated protein; BCRP: breast cancer resistance protein; SLC: solute carrier; ABC: ATP-binding cassette.

Transporter Family	Transporter	Main Expression	Main Type of Substrates
*SLCOs*	OATP1A2 (*SLCO1A2*)	Ubiquitous	Organic anions
	OATP1B1 (*SLCO1B1*)	Liver	Organic anions
	OATP1B3 (*SLCO1B3*)	Liver	Organic anions
	OATP2B1 (*SLCO2B1*)	Liver, intestine	Organic anions
*SLC10A*	NTCP (*SLC10A1*)	Liver	Bile acids
*SLC15A*	PEPT1 (*SLC15A1*)	Intestine	Peptides
	PEPT2 (*SLC15A2*)	Kidney	Peptides
*SLC22A*	OCT1 (*SLC22A1*)	Liver	Organic cations
	OCT2 (*SLC22A2*)	Kidney	Organic cations
	OCTN1 (*SLC22A*4)	Kidney	Organic cations/carnitine
	OCTN2 (*SLC22A*5)	Kidney	Organic cations/carnitine
	OAT1 (*SLC22A6*)	Kidney	Organic anions
	OAT2 (*SLC22A7*)	Liver	Organic anions
	OAT3 (*SLC22A8*)	Kidney	Organic anions
	OAT4 (*SLC22A11*)	Kidney, placenta	Organic anions
*SLC28A*	CNT1 (*SLC28A*1)	Kidney, liver, intestine	Nucleosides
	CNT2 (*SLC28A2*)	Ubiquitous	Nucleosides
	CNT3 (*SLC28A3*)	Ubiquitous	Nucleosides
*SLC29A*	ENT1 (*SLC29A1*)	Ubiquitous	Nucleosides
	ENT2 (*SLC29A2*)	Ubiquitous	Nucleosides
*SLC47A*	MATE1 (*SLC47A1*)	Liver, kidney	Organic cations
	MATE2-K (*SLC47A2*)	Kidney	Organic cations
*ABCB*	P-gp (*ABCB1*)	Intestine, liver, kidney, blood-brain barrier	Hydrophobic compounds
	BSEP (*ABCB11*)	Liver	Bile acids
*ABCC*	MRP1 (*ABCC1*)	Ubiquitous	Hydrophobic compounds, hydrophilic anions, conjugates
	MRP2 (*ABCC2*)	Intestine, liver, kidney	Hydrophilic anions, conjugates
	MRP3 (*ABCC3*)	Liver, kidney	Hydrophilic anions, conjugates
	MRP4 (*ABCC4*)	Liver, kidney, blood-brain barrier	Nucleotides
	MRP5 (*ABCC5*)	Ubiquitous	Nucleotides
*ABCG*	BCRP (*ABCG2*)	Intestine, liver, kidney, blood-brain barrier, stem cells	Hydrophobic compounds, hydrophilic anions, conjugates

**Table 2:** Classification of protein kinase C (PKC) isoforms.

Class	Dependence		Isoform
	Calcium	Diacylglycerol	
Classical/Conventional cPKC (cPKC)	Yes	Yes	PKCα
			PKCβ1
			PKCβ2
			PKCγ
Novel PKC (nPKC)	No	Yes	PKCδ
			PKCε
			PKCη
			PKCθ
Atypical PKC (aPKC)	No	No	PKCζ
			PKCλ/ι

**Table 3:** Main effects of the reference protein kinase C (PKC) activator phorbol-12-myristate-13-acetate (PMA) on drug transporter activity, localization and/or expression. P-gp: P-glycoprotein; BSEP: bile salt export pump; MRP: multidrug resistance-associated protein; BCRP: breast cancer resistance protein; OATP: organic anion transporting polypeptide; OAT: organic anion transporter; NTCP: sodium-taurocholate co-transporting polypeptide; OCT: organic cation transporter; OCTN: organic cation transporter novel; ENT: equilibrative nucleoside transporter; PEPT: peptide transporter.

Transporter	Activity	Localization	Expression
P-gp	Increase (human cancer cell lines, mouse renal proximal tubules) [13,59,60,61]		Increase (human cancer cells and primary human hepatocytes) [17,62,63]
BSEP		Internalization (rat liver) [64]	Decrease (primary human hepatocytes) [18]
MRP2		Internalization (human hepatic HepG2 cell line) [65]	No change (primary human hepatocytes) [18]
MRP3			Increase (primary human hepatocytes) [18]
BCRP			No change (primary human hepatocytes) [18]
OATP1A2		Internalization (OATP1A2- COS-7 cells) [66]	
OATP1B1		Internalization (OATP1B1-HEK293 cells) [67]	Decrease (primary human hepatocytes) [18]
OATP1B3	Decrease (primary human hepatocytes) [14]		Decrease (primary human hepatocytes) [18]
OATP2B1		Internalization (OATP2B1-MDCKII cells, Caco-2 cell line, human placenta, human hepatic HepaRG cell line) [68], (Figure 2a)	Decrease (primary human hepatocytes) [18]
OAT1		Internalization (OAT1-COS-7 cells) [69]	
OAT3	Decrease (rabbit renal proximal tubules) [70]		
NTCP		Internalization (primary rat hepatocytes, NTCP-HepG2 cells) [64,71]	Decrease (primary human hepatocytes) [18]
OCT1		No change (Figure 2b)	Decrease (primary human hepatocytes) [18]
OCTN2		Increase in membrane expression (rat astrocytes) [72]	
ENT1	Increase (ENT1-PK15-NTD cells) [73]	Increase in membrane expression (ENT1- PK15-NTD cells) [73]	
PEPT1	Decrease (human intestinal Caco-2 cell line) [74]		
PEPT2	Decrease (porcine kidney LLC-PK1 cell line) [75]		

**Table 4:** Examples of new molecular entities targeting protein kinases C (PKCs). FLT3: Fms-like tyrosine kinase 3; cPKCs: classical/conventional PKCs; nPKCs: novel PKCs.

Drug	Nature of Effect	Targeted PKC(s)	Putative Therapeutic Indication
Rubixostaurin	PKC inhibition	PKCβ	Microvascular complications of diabetes [163,164]
Enzastaurin	PKC inhibition	PKCβ	Cancers [165]
Tamoxifen	PKC inhibition	Pan-PKC	Bipolar disorders [166]
Sotrastaurin (AEB071)	PKC inhibition	Pan-PKC	Organ transplantation [167], psoriasis [168]
KAI-9803	PKC inhibition	PKCδ	Coronary intervention for myocardial infarction [169]
Midostaurin	PKC/FLT3/multikinase inhibition	Pan-PKC	Leukemias [170]
Ingenol mebutate	PKC activation	cPKCs/nPKCs	Actinic keratoses [171]

## Abbreviations

PKC	Protein kinase
mRNA	Messenger ribonucleic acid
cPKC	Classical/conventional PKC
nPKC	Novel PKC
aPKC	Atypical PKC
DAG	Diacylglycerol
SLC	Solute carrier
ABC	ATP-binding cassette
OATP	Organic anion transporting polypeptide
OAT	Organic anion transporter
OCT	Organic cation transporter
NTCP	Sodium-taurocholate co-transporting polypeptide
MATE	Multidrug and toxin extrusion protein
OCTN	Organic cation/carnitine transporter
PEPT	Peptide transporter
CNT	Concentrative nucleoside transporter
ENT	Equilibrative nucleoside transporter
P-gp	P-glycoprotein
MRP	Multidrug resistance-associate protein
BCRP	Breast cancer resistance protein
BSEP	Bile salt export pump
PMA	Phorbol myristate acetate
BIM	Bisindolylmaleimide
PK15-NTD	Pig kidney epithelial nucleoside transporter deficient
cAMP	3′,5′-cyclic adenosine monophosphate
